# Supplementary material for: Fine-mapping from summary data with the “Sum of Single Effects” model
Source: PLoS Genet. 2022 Jul 19;18(7):e1010299. doi: 10.1371/journal.pgen.1010299 (PMC9337707; doi:10.1371/journal.pgen.1010299)
Supplement: S1 Fig — These plots summarize the likelihood ratios LRj for SNPs j in simulated fine-mapping data sets, separately for allele-flip SNPs with an effect (top row, right-hand side), without an effect on the trait (top row, left-hand side), and for SNPs without a flipped allele that affect the trait (middle row, right-hand side) and do not affect the trait (middle row, left-hand side). The two histograms in the bottom row show likelihood ratios after restricting to SNPs with z-scores greater than 2 in magnitude. The bar heights in the histograms in the middle and bottom rows are drawn on the logarithmic scale to better visualize the smaller numbers of SNPs with likelihood ratios greater than 1 (i.e., log LRj > 0). (PDF) [file pgen.1010299.s001.pdf]

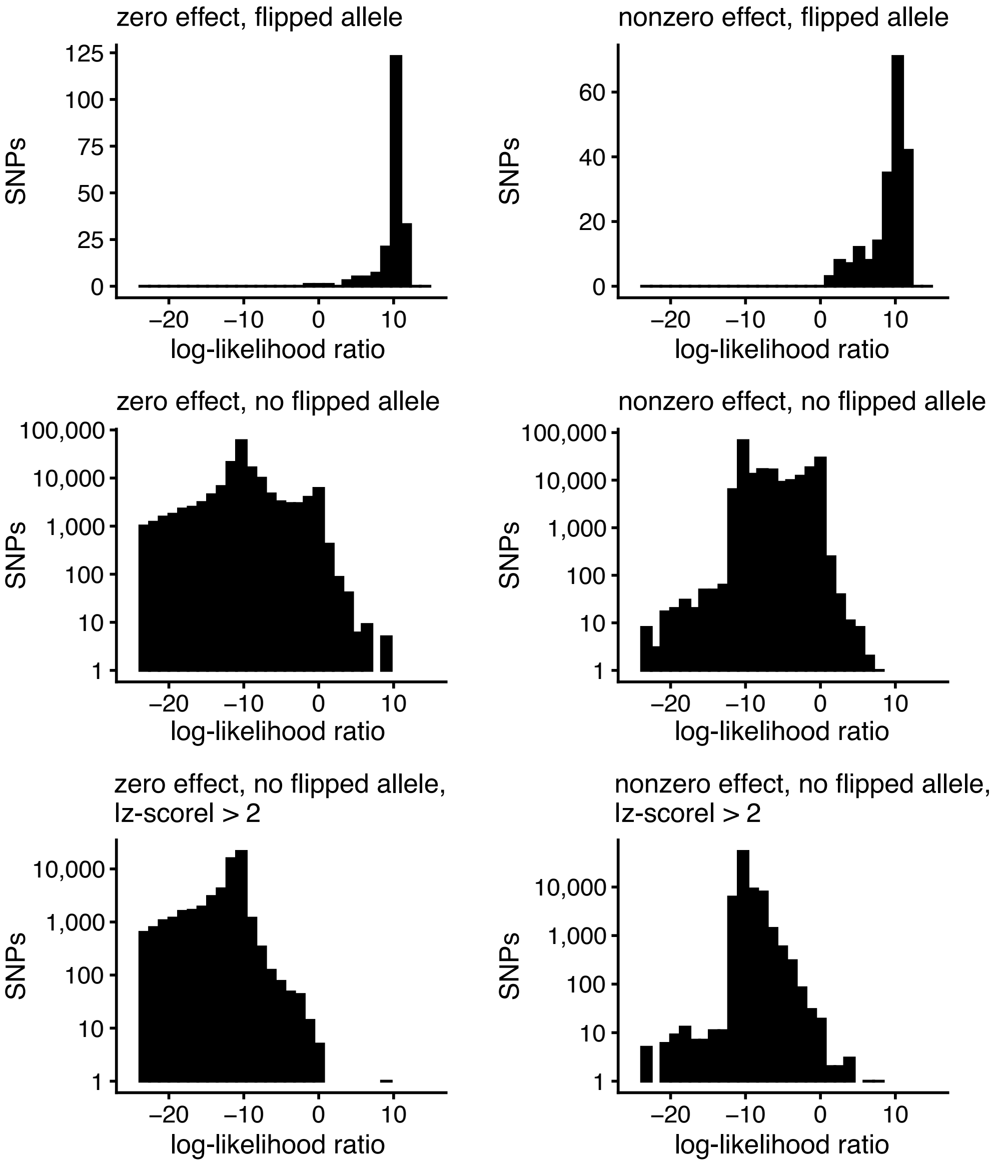

zero effect, flipped allele
nonzero effect, flipped allele
zero effect, no flipped allele
nonzero effect, no flipped allele
zero effect, no flipped allele, |z-score| > 2
nonzero effect, no flipped allele, |z-score| > 2
SNPs
log-likelihood ratio
125
100
75
50
25
0
60
40
20
100,000
10,000
1,000
100
10
1
−20
−10
0
10
