# Supplementary material for: Fine-mapping from summary data with the “Sum of Single Effects” model
Source: PLoS Genet. 2022 Jul 19;18(7):e1010299. doi: 10.1371/journal.pgen.1010299 (PMC9337707; doi:10.1371/journal.pgen.1010299)
Supplement: Attachment — Submitted filename: response_to_referees.pdf [file pgen.1010299.s003.pdf]

RESPONSE TO REVIEWS AND SUMMARY OF THE REVISIONS

By Yuxin Zou[1], Peter Carbonetto[2] Gao Wang[3] and Matthew Stephens[4]

[1]*Department of Statistics, University of Chicago, Chicago, IL, USA, yuxin@uchicago.edu*

[2]*Department of Human Genetics and the Research Computing Center, University of Chicago, Chicago, IL, USA, pcarbo@uchicago.edu*

[3]*Department of Neurology and the Gertrude. H. Sergievsky Center, Columbia University, New York, NY, USA, wang.gao@columbia.edu, wang.gao@columbia.edu*

[4]*Departments of Statistics and Human Genetics, University of Chicago, Chicago, IL, USA, mstephens@uchicago.edu*

We thank the reviewers for their detailed feedback on our manuscript, "Fine-mapping from summary data with the 'Sum of Single Effects' model." The reviewers' feedback, and in particular the feedback from Reviewer 2, prompted us to reconsider large parts of the presentation. As a result our resubmission differs in many places from our initial submission (places in the text that have been substantially revised are in blue). We summarize the main changes below.

We note that the core of our new fine-mapping methods did not fundamentally change—the main change is a modification to better deal with the "high PVE setting" raised by Reviewer 2. However, we hope that the new presentation is simpler and provides a better understanding of existing fine-mapping methods, why they work well (or not), and under what circumstances. For example, the new presentation better highlights the fact that some methods use *adjusted* z-scores (e.g., SuSiE-RSS, FINEMAP) and others do not (e.g., DAP-G, CAVIAR), and since the unadjusted z-scores introduce an additional approximation that is violated when effect sizes are large, in experiments we see that SuSiE-RSS and FINEMAP perform much better than the other methods in these circumstances. We believe that the resulting manuscript is substantially improved over our initial submission, and we hope the reviewers agree with this. We also wish to thank the reviewers for their patience as we worked to address their comments.

Several improvements were made to the manuscript to address, directly or indirectly, the reviewers' comments. We summarize the main changes here:

1. We modified our *SuSiE-RSS* method to better deal with the high PVE setting. Apart from improving performance in high-PVE setting, this modification has a conceptual benefit (even in the low-PVE setting): it makes *SuSiE-RSS*, when used with an in-sample LD matrix, exactly equivalent to SuSiE on the individual data. As a result, we removed the *SuSiE-suff* terminology, since this is now just a special case *SuSiE-RSS*. We added a new table (Table 1) to summarize these ideas, and to help readers understand the relationships among the methods.

2. This modification also led to an improved understanding of connections and differences between fine-mapping methods for summary data, particularly in the special case when $\boldsymbol{X}, \boldsymbol{y}$ are standardized (see Section, "Special case when $\boldsymbol{X}, \boldsymbol{y}$ are standardized"). In this special case (which is also a very common case in GWAS), as noted by Benner et al. (2018), one can replace $\boldsymbol{X}^\intercal \boldsymbol{y}$ with $\sqrt{N}\tilde{\boldsymbol{z}}$, where N is the sample size and $\tilde{\boldsymbol{z}}$ is the vector of "PVE-adjusted z-scores." A key difference among existing fine-mapping methods can then be framed as whether or not they use the PVE-adjusted z-scores (see "Connections with previous work").

3. We included a new set of experiments to evaluate the performance of fine-mapping methods in the high-PVE setting (addressing a comment by Reviewer 2). See “Fine-mapping causal SNPs with larger effects” in Results.
4. We improved the figures, in particular Fig 3 and Fig 5, to make the results more clear and more comparable across panels (addressing a comment by Reviewer 3).
5. We performed an empirical assessment of the likelihood-ratio statistic for identifying allele flips (addressing a comment by Reviewer 1). See “Likelihood ratio for detecting allele flips” in S1 Text.
6. Finally, we improved and simplified the `susie_rss` interface in the `susieR` R package.

Comments from Reviewer 1. Zou *et al* present their work that extends the sum of single effects (SuSiE) Bayesian sparse model for fine-mapping to operate directly on GWAS summary statistics. To do so, they describe the sufficient statistics for fine-mapping, which happen to be marginal effect-size estimates and linkage disequilibrium information (LD). They demonstrate that this setting can accommodate either effect-size estimates along with standard errors, or z-score statistics, which are much more commonly released in GWAS data. In addition to this extension, Zou *et al* describe approaches to perform inference in a low-rank setting, which is commonly the case when estimating LD from reference population data (e.g., 1000 Genomes). Lastly, Zou *et al* also describe approaches to QC summary data under model assumptions and identify SNPs with improperly labeled encoding (*i.e.*, “allele flips”). The manuscript presents simulated data results to support their claims, is well written, and easy to follow. SuSiE has quickly become a standard approach for fine-mapping in recent years and I was excited to see the authors describe a robust summary-based version. I have some comments below.

Major Comments: The main exposition of the SuSiE-suff and SuSiE-RSS approaches is very well done, justified by empirical results and I have no major comments to include regarding the primary fine-mapping procedure.

Minor Comments: The authors present a nice strategy to QC allele flips in summary data using a likelihood ratio test under a mixture model. Their approach is well described, and the authors provide some guidelines on how to use this in practice. The authors are clear in that this procedure should not be considered as an automated approach to QC and should be done interactively. Given that, I would still appreciate to see some Q-Q plot (or something similar) to see how well the fitted LRTs behave under the null and its power under the alternate. Results in this setting could further justify the interactive nature for this tool.

Response to Reviewer 1. We thank the reviewer for these comments.

In response to the comment about identifying allele flips, in the revised manuscript we added simulations to evaluate the use of the likelihood-ratio statistic. These simulations are described in S1 Text (“Likelihood ratio for detecting allele flips”), and the results are summarized in Fig 7.

To clarify, the likelihood ratio we compute is a likelihood ratio, and *not* the commonly-used “likelihood-ratio test statistic” for testing nested hypotheses. We mistakenly used the word “test” at a couple of points in our original submission; we apologize for this and have removed these occurences.

Comments from Reviewer 2. The paper presents a version of the fine-mapping model SuSiE that is applicable to summary data (z-scores and LD matrix) while the previously published SuSiE model was applicable to individual level data (genotypes and phenotypes). Additionally, paper considers three topics related to practical issues of fine-mapping: (1)

detecting inconsistencies between z-scores and LD estimates, (2) regularizing the estimated LD matrix, and (3) a computational refinement procedure of SuSiE algorithm. As examples the study uses simulated phenotypes on UK Biobank genetic data.

Paper is well-written and references to existing work are appropriate. Since SuSiE is a key method for fine-mapping, its implementation applicable to summary data is an important contribution to the field and it is/will be used widely. However, when considering the guidelines for publications in this forum, it is less clear what in this paper presented "a new way of approaching a biological or biomedical question, or a substantive advance over existing approaches." First, there are well-established fine-mapping methods (such as FINEMAP and DAP-G) that work with the same idea (replace $\mathbf{X}^T\boldsymbol{y}$ by scaled effect estimates and $\mathbf{X}^T\mathbf{X}$ by scaled LD matrix in linear model likelihood) which produce similar results as SuSiE. Second, the proposed detection of inconsistencies between z-scores and LD matrix seems similar to recently published DENTIST method (Chen et al., 2021), which is also stated in this paper. Third, while, to my knowledge, these kind of LD matrix regularization results have not been presented before in fine-mapping context, and therefore they are interesting, the regularization approach does not seem that important in practice. Fourth, the computational refinement procedure of SuSiE algorithm is a technical fix to SuSiE algorithm rather than a considerable improvement over the existing methods.

Major comments:

Hat-notation for $\boldsymbol{b}$ *and* $\boldsymbol{z}$. I find it confusing that paper defines vector $\boldsymbol{b}$ as *multiple* regression coefficient but uses $\hat{\boldsymbol{b}}$ as an estimate for coefficients from *simple* regression. Thus, in this paper, $\hat{\boldsymbol{b}}$ is not an estimate of $\boldsymbol{b}$. This is against common statistical notation and therefore likely causes confusion for a very large group of readers. I would suggest using the standard notation where hat denotes an estimate of the very parameter on which the hat is put. Same comment about z-scores.

Assumption about effects being very small. Line 172 states that model *RSS-Z* is valid only when all non-zero effects are very small. While this indeed is the most common case, there are also loci that explain several percentages of variance and typically these are highly interesting loci for fine-mapping with multiple causal variants in them. Does SuSiE model handle these loci correctly when applied to individual level data? And what about summary data? If you ran your simulated examples with variance explained set to 30% instead of 0.5% what would happen with each method? Please include in the manuscript some clear example or statement about this. (At least FINEMAP should handle appropriately such cases https://doi.org/10.1101/318618)

Minor comments:

l. 87, "tractible" should be "tractable".

l. 94 "Approximate posterior of $\boldsymbol{b}_1, \ldots, \boldsymbol{b}_L$": Doesn't the algorithm converge to only one of the $L!$ symmetrical modes of the posterior rather than to the actual posterior?

l. 121 Says that sufficient statistics contain "exactly the same information as individual level data." More precisely they contain same information about the parameters of a particular model considered here. But they don't, in general, contain "exactly the same information" as full data.

l. 122 The statistics mentioned are sufficient statistics for the parameters of the SuSiE model. (They are not any general "sufficient statistics" of these data.)

l. 233 It is unclear what "refine" means here? Is it "rerun until convergence starting from the current state"?

l. 289 Remove extra "care".

l. 373. Say that samples have self-reported their ethnicity as white British. (If that is indeed the case.)

Figure 2 legend defines "power" and "FDR" that are then used also in other Figures. Would be better to define these in text once and then use in all Figures.

l. 497 Benner et al. (2016) suggest how to interpret the linear model parameters to account for properties of binary data such as case-control ratio.

l. 669 "Then all $R_{jj'}$ do not appear in the likelihood." Do you mean "then none of $R_{jj'}$ appear in the likelihood"?

l. 677–678 "rows and columns in γ." Do you mean the square submatrix of dimension $|\gamma|$ of $\hat{\boldsymbol{R}}$ formed by subrows and subcolumns corresponding to elements in γ? Similarly for $-\gamma$.

l. 712. The fact that no LD is needed to do proper inference is also true for binary traits as stated already by Maller et al. (2012). See Supp Text p. 56–57 of Maller et al. (2012).

l. 773. Hard to believe that the above mentioned criteria result in exactly 50,000 samples. Make clear how you end up with exactly 50,000?

Response to Reviewer 2. We thank the reviewer for their thorough and detailed feedback. We were pleased that the reviewer recognized that the extension of SuSiE to summary data is "an important contribution to the field" and "is/will be widely used." While the referee is correct that there are other existing fine-mapping approaches that use summary data and produce similar results to SuSiE, the SuSiE approach has both computational and qualitative advantages (already explored extensively in the original SuSiE paper, and so not repeated here), and we believe that its extension to summary data therefore represents the kind of "substantive advance" that merits publication in *PLoS Genetics*. In addition, the paper contains several new results; for example, we discover the that the two most competitive methods, FINEMAP and SuSiE-RSS, tend to overestimate the number of causal SNPs with a misspecified LD matrix. And while the DENTIST work is based on similar ideas to ours, the two approaches were developed independently, and differ in detail; for example, DENTIST makes use of the pseudoinverse that we demonstrate in our paper to be undesirable, and does not provide the kinds of likelihood ratio statistic that we have proposed. We hope that our improvements to the manuscript, together with a new, more unified reassessment of fine-mapping methods, will persuade the reviewer of the suitability of our work for *PLoS Genetics*.

The reviewer's second major comment ("Assumption about effects being very small") inspired us to rethink aspects of SuSiE-RSS, and lead us to develop a better, more cohesive understanding of fine-mapping methods for summary data. A key idea, borrowing from Benner et al. (2018), was to treat more carefully the special case when $\boldsymbol{X}, \boldsymbol{y}$ are standardized. In this special case (which is also a very common case in GWAS), one can replace $\boldsymbol{X}^\intercal \boldsymbol{y}$ with $\sqrt{N}\tilde{\boldsymbol{z}}$, where N is the sample size and $\tilde{\boldsymbol{z}}$ is the vector of "PVE-adjusted z-scores." This simple idea is what allowed us to more clearly draw connections among existing fine-mapping methods. (Note that computing the PVE-adjusted z-scores requires knowledge of N, and it seems reasonable to assume that this is available in most cases.) In particular, we concluded,

> When $\hat{\boldsymbol{R}}$ is invertible, these previous approaches (Benner et al., 2016; Hormozdiari et al., 2014; Kichaev et al., 2014; Lee et al., 2018) are the same as our approach except that they use the z-scores, $\hat{\boldsymbol{z}}$, instead of the PVE-adjusted z-scores, $\tilde{\boldsymbol{z}}$. Thus, where our approach uses the identity $\boldsymbol{X}^\intercal \boldsymbol{y} = \sqrt{N}\tilde{\boldsymbol{z}}$, these previous approaches are implicitly making the approximation $\boldsymbol{X}^\intercal \boldsymbol{y} \approx \sqrt{N}\hat{\boldsymbol{z}}$. If all effect sizes are small (*i.e.*, PVE ≈ 0), then $\tilde{\boldsymbol{z}} \approx \hat{\boldsymbol{z}}$, and the approximation will be nearly exact; on the other hand, if the PVE is not close to zero for some SNPs, then the use of the PVE-adjusted z-scores is preferred (Benner et al., 2018).

Also, as suggested by the reviewer, we have included additional experiments to compare the performance of fine-mapping methods when the SNPs explain a larger proportion of variance in the trait. These experiments confirmed our expectation that methods such as DAP-G and CAVIAR that (implicitly) make the approximation $\boldsymbol{X}^\intercal \boldsymbol{y} \approx \sqrt{N}\hat{\boldsymbol{z}}$ perform much worse when the SNP effects are large. These experiments generated other useful insights and we invite the reviewer to examine them (see "Fine-mapping causal SNPs with larger effects" in the manuscript).

Regarding the "hat notation" used, this is notation used in previous publications, e.g., Stephens (2016); Zhu and Stephens (2017). However, we agree that the notation is probably not familiar to many readers, and may be a source of confusion; in the revised manuscript, we have rewritten some of the likelihood expressions so that they do not include both the multiple regression coefficients, $\boldsymbol{b}$, and the marginal association statistics, $\hat{\boldsymbol{b}}$. More generally, we have substantially revised the presentation of the methods to better clarify assumptions and draw connections to existing methods, which we hope will also address confusion with the notation.

Regarding DENTIST, we agree that the ideas proposed in that paper are similiar to ours, and we are now more careful to cite Chen et al. (2021), and draw connections. However, there are some important differences which we now highlight in the manuscript: first, DENTIST uses the z-scores whereas we recommend using the "PVE-adjusted" z-scores; second, DENTIST replaces the inverse of the LD matrix, $\hat{\boldsymbol{R}}$, with its pseudoinverse, which, as we caution, may lead to undesirable behaviour (see "Fine-mapping with inconsistent summary data and a non-invertible LD matrix: an illustration"); third, the current implementation of DENTIST (available at `https://github.com/Yves-CHEN/DENTIST`) requires both summary data and individual-level data, whereas our implementation (the function `kriging_rss` in `susieR`) runs using only summary data ($\boldsymbol{z}, \hat{\boldsymbol{R}}, N$).

Regarding regularization of the LD matrix, we agree that this is not a central contribution of the paper. Nonetheless, a surprising result was that the performance of some methods, notably FINEMAP, was sensitive to the choice of regularization, and in some cases estimating the regularization parameter greatly improved FINEMAP's performance.

To respond to the reviewer's question about the IBSS algorithm ("Doesn't the algorithm converge to only one of the $L!$ symmetrical modes of the posterior rather than to the actual posterior?"), we did not elaborate on the IBSS algorithm, nor the variational approximation, since these are not essential to explaining the main contributions of the paper. Instead, we referred the reader to Wang et al. (2020) for details. The variational approximation assumes that the single effects $\boldsymbol{b}_1, \ldots, \boldsymbol{b}_L$ are conditionally independent. This is what makes the computations tractable. But, as a result of this constraint, the approximate posterior is necessarily an approximation (except when $L = 1$). As the reviewer rightly notes, this approximate posterior, due to the conditional independence assumption, cannot capture the (trivial) symmetric modes of the posterior. But this is arguably a trivial problem since we rarely care about the symmetric modes. A less trivial issue is that there can be many other locally optimal approximate posteriors. (This issue is discussed in Wang et al. 2020, and has beenn discussed in other papers on variational inference.) In general, we can only guarantee that the IBSS algorithm will converge to a local optimum (except, again, when $L = 1$). The refinement procedure described in our paper was an attempt to address this problem in cases where the local optimum provides a particularly poor fit.

To illustrate, we implemented a toy example in R in which 4 variables affect the outcome y. When L, the number of single effects, is incorrectly set to $L = 2$, the IBSS algorithm will settle on different approximate posteriors depending on how the algorithm is initialized:

```
library(MASS)
library(susieR)
set.seed(1)
n = 400
p = 20
s = 0.8
beta = rep(0,p)
beta[1:4] = 1
S = s^abs(outer(1:p,1:p,"-"))
X = mvrnorm(n,rep(0,p),S)
```

```
X = scale(X,center = TRUE,scale = TRUE)
y = drop(X %*% beta + rnorm(n))
fit1 = susie(X,y,L = 2s_init = susie_init_coef(c(1,2),c(1,1),p))
fit2 = susie(X,y,L = 2,s_init = susie_init_coef(c(3,4),c(1,1),p))
unlist(fit1$sets$cs)
unlist(fit2$sets$cs)
packageVersion("susieR")
# L1 L2
#  3  1
# L1 L2
#  2  4
# 0.11.96
```

In the Discussion, we briefly discussed how to extend these fine-mapping ideas to binary or case-control traits. To this point, the reviewer said, "Benner et al. (2016) suggest how to interpret the linear model parameters to account for properties of binary data such as case-control ratio." Our understanding is that Benner et al. (2016) is referring a result given in the supplementary materials of Pirinen, Donnelly and Spencer (2013), in particular equation (1.6). However, it seems that Pirinen *et al* define $\hat{\beta}$ to be the maximum-likelihood estimate (MLE) in the multiple regression, not the vector of MLEs from the univariate regressions, so it isn't clear to us how their result applies to the summary-data setting. But perhaps we have misinterpreted this comment and if so we would welcome a clarification.

The ethnicity of the UK Biobank individuals is not self-reported; this is based on PCA. We have clarified this in the text.

We also thank the reviewer for highlighting several mistakes, typos and confusing or ambiguous statements made in the manuscript. We have corrected these errors in the revised submission. We have also cited Maller et al. (2012) as suggested.

Comments from Reviewer 3. This manuscript describes the extension of the recently proposed SuSiE model to summary data (z-scores and correlation matrix), extending its applicability to fine mapping for genetic summary data.

The manuscript is clearly written, and the mathematical exposition is careful and sufficiently detailed to follow.

Inconsistency between summary estimates and the LD matrix is an important problem which can produce misleading results and slow convergence. A good section of the manuscript is dedicated to dealing with these, through regularization of the LD matrix, including estimating the regularization parameter λ, which I think is novel. Detecting inconsistencies though is a thorny problem, and a new method is proposed for those. However, the computational complexity is high, perhaps higher than fitting the SuSiE model itself. And so some guidance about when this should be considered would be useful. Are there diagnostics from the SuSiE output that indicate something could be awry and suggest it would be worth running the discrepancy detection? For example, in my own experience finding multiple credible sets (up to 10) often containing only one SNP, or containing SNPs with no marginal evidence for association has indicated issues with the data.

Overall, this is an important contribution, extending the use of the new SuSiE approach to summary data. The approach is already in widespread use in the statistical genetics and bioinformatics communities, so the exposition of the underlying mathematics and associated comments on how the approach should be applied is very timely.

Minor comments: In Fig 4 [now Fig 5], I would like to compare SuSiE-RSS to other approaches, but as each approach has its own subplot and there are no grid lines, this is very hard to do. Could the results be faceted data (LD sample size, lambda, *etc*) or grid lines added? The dotted black line is there in each plot, but is not enough for me to decide whether the green lines in D are above/below the green lines in F, for example.

Response to Reviewer 3. We thank the reviewer for their constructive comments and suggestions.

As the reviewer noted, we have also found that SuSiE can behave unpredictably when the LD matrix is inconsistent with the z-scores. In the original manuscript, we wrote, "Anecdotally, we have found large inconsistencies like these often cause SuSiE to converge very slowly and produce misleading results such as an unexpectedly large number of CSs." Since this is mainly anecdotal, we are hesistant to add much more detail without investigating this more systematically. In the revised manuscript, we expanded on this statement slightly: "Anecdotally, we have found large inconsistencies like these often cause SuSiE to converge very slowly and produce misleading results, such as an unexpectedly large number of CSs, or two CSs containing SNPs that are in strong LD with each other."

Finally, following the reviewer's suggestion, we have reworked Fig 3 and Fig 5 to make the results more comparable across the different panels.

REFERENCES

BENNER, C., SPENCER, C. C. A., HAVULINNA, A. S., SALOMAA, V., RIPATTI, S. and PIRINEN, M. (2016). FINEMAP: efficient variable selection using summary data from genome-wide association studies. *Bioinformatics* **32** 1493–1501.

BENNER, C., HAVULINNA, A. S., SALOMAA, V., RIPATTI, S. and PIRINEN, M. (2018). Refining fine-mapping: effect sizes and regional heritability. *bioRxiv* **doi:10.1101/318618**.

CHEN, W., WU, Y., ZHENG, Z., QI, T., VISSCHER, P. M., ZHU, Z. and YANG, J. (2021). Improved analyses of GWAS summary statistics by reducing data heterogeneity and errors. *Nature Communications* **12** 7117.

HORMOZDIARI, F., KOSTEM, E., KANG, E. Y., PASANIUC, B. and ESKIN, E. (2014). Identifying causal variants at loci with multiple signals of association. *Genetics* **198** 497–508.

KICHAEV, G., YANG, W.-Y., LINDSTROM, S., HORMOZDIARI, F., ESKIN, E., PRICE, A. L., KRAFT, P. and PASANIUC, B. (2014). Integrating functional data to prioritize causal variants in statistical fine-mapping studies. *PLoS Genetics* **10** e1004722.

LEE, Y., LUCA, F., PIQUE-REGI, R. and WEN, X. (2018). Bayesian multi-SNP genetic association analysis: control of FDR and use of summary statistics. *bioRxiv* **doi:10.1101/316471**.

MALLER, J. B., MCVEAN, G., BYRNES, J., VUKCEVIC, D., PALIN, K., SU, Z., HOWSON, J. M. M., AUTON, A., MYERS, S., MORRIS, A., PIRINEN, M., BROWN, M. A., BURTON, P. R., CAULFIELD, M. J., COMPSTON, A., FARRALL, M., HALL, A. S., HATTERSLEY, A. T., HILL, A. V. S., MATHEW, C. G., PEMBREY, M., SATSANGI, J., STRATTON, M. R., WORTHINGTON, J., CRADDOCK, N., HURLES, M., OUWEHAND, W., PARKES, M., RAHMAN, N., DUNCANSON, A., TODD, J. A., KWIATKOWSKI, D. P., SAMANI, N. J., GOUGH, S. C. L., MCCARTHY, M. I., DELOUKAS, P., DONNELLY, P. and CONSORTIUM, T. W. T. C. C. (2012). Bayesian refinement of association signals for 14 loci in 3 common diseases. *Nature Genetics* **44** 1294–1301.

PIRINEN, M., DONNELLY, P. and SPENCER, C. C. A. (2013). Efficient computation with a linear mixed model on large-scale data sets with applications to genetic studies. *Annals of Applied Statistics* **7** 369–390.

STEPHENS, M. (2016). False discovery rates: a new deal. *Biostatistics* **18** kxw041.

WANG, G., SARKAR, A., CARBONETTO, P. and STEPHENS, M. (2020). A simple new approach to variable selection in regression, with application to genetic fine mapping. *Journal of the Royal Statistical Society, Series B* **82** 1273–1300.

ZHU, X. and STEPHENS, M. (2017). Bayesian large-scale multiple regression with summary statistics from genome-wide association studies. *Annals of Applied Statistics* **11** 1561–1592.
