# Supplementary material for: Fine-mapping from summary data with the “Sum of Single Effects” model
Source: PLoS Genet. 2022 Jul 19;18(7):e1010299. doi: 10.1371/journal.pgen.1010299 (PMC9337707; doi:10.1371/journal.pgen.1010299)
Supplement: Attachment — Submitted filename: response_to_referees.pdf [file pgen.1010299.s004.pdf]

RESPONSE TO REVIEWS AND SUMMARY OF THE REVISIONS

By Yuxin Zou[1], Peter Carbonetto[2] Gao Wang[3] and Matthew Stephens[4]

[1]*Department of Statistics, University of Chicago, Chicago, IL, USA, yuxin@uchicago.edu*

[2]*Department of Human Genetics and the Research Computing Center, University of Chicago, Chicago, IL, USA, pcarbo@uchicago.edu*

[3]*Department of Neurology and the Gertrude. H. Sergievsky Center, Columbia University, New York, NY, USA, wang.gao@columbia.edu, wang.gao@columbia.edu*

[4]*Departments of Statistics and Human Genetics, University of Chicago, Chicago, IL, USA, mstephens@uchicago.edu*

In this letter, we are responding to Reviewer 2's comments regarding FINEMAP's performance in the "high PVE" setting. We have added some additional explanation to accompany these results in the manuscript; please see "Fine-mapping causal SNPs with larger effects", where the last paragraph, highlighted in blue, has been revised.

Comments from Reviewer 2. I thank the authors for their additional work related to adopting adjusted z-scores and running high PVE simulations and for their detailed answers to previous comments.

These new results pose one more question, namely why FINEMAP performs so much worse than SuSiE with increasing PVE when both should handle the situation through the same adjusted z-scores and when Benner et al. (2018) did not observe problems with FINEMAP in high PVE cases. To understand this, it would be useful to know whether the FINEMAP problem in high PVE cases appear only with approximate LD matrix (currently shown results) or also with in-sample LD matrix (not currently shown).

In new simulations, N has been considerably dropped from previous simulations. As N now varies across simulations, please cross-check once more that all methods are always run with the correct value for N.

Response to Reviewer 2. Thank you for this additional feedback. We investigated this result more closely in "Fine-mapping causal SNPs with larger effects."

First, we confirmed that FINEMAP works very well with an in-sample LD matrix in these high-PVE settings (results not shown); the drop in performance only occurs with an out-of-sample LD matrix. A partial explanation for FINEMAP's much worse performance with out-of-sample LD was that FINEMAP often overestimated the number of causal SNPs; in 17% of the 30%-PVE simulations, FINEMAP assigned highest probability to configurations with more causal SNPs than the true number. By contrast, SuSiE-RSS overestimated the number of causal SNPs (the number of CSs) in only 1% of the simulations.

Second, we noted that this drop in performance was much more acute when FINEMAP attempted to estimate the number of causal SNPs. So we suggested a simple remedy by fixing the number of causal SNPs to be small.

Finally, we created a short vignette, available at `https://stephenslab.github.io/finemap/large_effect.html`, which may be helpful to you and others for verifying this result. In this example, SuSiE produces a nearly identical result when provided with either the in-sample or out-of-sample LD matrix. FINEMAP works as expected with the in-sample LD matrix, but produces unexpectedly poor results with the out-of-sample LD matrix.

REFERENCES

BENNER, C., HAVULINNA, A. S., SALOMAA, V., RIPATTI, S. and PIRINEN, M. (2018). Refining fine-mapping: effect sizes and regional heritability. *bioRxiv* **doi:10.1101/318618**.
